# Exploring healthcare students’ interprofessional teamwork in primary care simulation scenarios: collaboration to create a shared treatment plan

**DOI:** 10.1186/s12909-021-02852-z

**Published:** 2021-08-03

**Authors:** Lene Lunde, Anne Moen, Rune B. Jakobsen, Elin O. Rosvold, Anja M. Brænd

**Affiliations:** 1 Department of Nursing Science, Institute of Health and Society, Faculty of Medicine, University of Oslo, Oslo, Norway; 2 Department of Health Management and Health Economics, Institute of Health and Society, Faculty of Medicine, University of Oslo, Oslo, Norway; 3 Department of General Practice, Institute of Health and Society, Faculty of Medicine, University of Oslo, Oslo, Norway

**Keywords:** Primary care, Interprofessional education, Simulation, Interaction, Healthcare students

## Abstract

**Background:**

Primary care providers assume responsibility for patients with increasingly complex problems requiring interprofessional collaboration. Introducing interprofessional education in healthcare curricula prepares healthcare students for this reality. Solving simulation scenarios as an educational strategy is promoted to support interprofessional education in health care, and is mostly used in acute clinical situations. This paper aims to explore how healthcare students’ actions influence interprofessional collaboration and treatment plan identification when they solve common, sub-acute patient scenarios in primary care situations.

**Methods:**

Interaction analysis of video recordings from the simulation scenarios was performed with a focus on the students’ joint actions; specifically how these actions unfold and how productive the students were in terms of developing treatment plans.

**Results:**

We found variation in the groups’ interactions, the paths they followed, and the quality of their knowledge output in their shared treatment plan. The groups with the capacity to collaborate and engage in sharing information, and explain and elaborate on concepts, were more successful in developing comprehensive treatment plans. Furthermore, these groups managed the duality of defining and solving the immediate problem and collaboratively preparing for future care.

**Conclusions:**

Analysis of the activities in our scenarios showed the students’ potential to practice interprofessional collaboration. Our study illustrates that simulation of sub-acute scenarios in primary care is an underexplored but suitable arena to train communication and teamwork in complex situations. The simulation scenarios are also feasible for use on-site in an educational facility or in practice with minimal equipment and resources.

## Background

Primary care professionals assume responsibility for patients with increasingly complex problems. Shorter hospital stays and increased emphasis on home care and aging in place suggest that more people will require primary health care [1]. To meet such new challenges and offer optimized quality patient care, working in interprofessional teams will be the preferred practice [2, 3]. Introducing interprofessional education (IPE) into healthcare curricula prepares healthcare students for interprofessional collaboration [3].

IPE implies that students from two or more professions engage in interactions to learn about, from, and with one another to improve collaboration and quality of care [1, 4]. Research has showed that students in healthcare IPE programs gained confidence, improved communication skills, adopted more positive attitudes towards interprofessional learning and team care, and enhanced their understanding of the roles of other professionals after participating in IPE [5, 6]. Despite broad consensus on the importance of IPE, there is no consensus on how to integrate IPE into healthcare education, or go beyond profession-specific teaching and overcome practical constrains such as schedules, actual space capacity, teacher resources, and economics [7, 8].

Healthcare education programs may have different perspectives on learning and teaching, adding to the barriers of implementing IPE. Based on the definition of IPE, where learning “about, from and with” one another is the cornerstone, we adopted a socio-cultural perspective to understand interprofessional learning. Facilitating IPE requires effective teaching methods, and the use of simulation as an educational strategy is promoted to support IPE in healthcare education [9, 10].

The use of simulation scenarios is recognized as a facilitator for active learning to develop clinical and collaborative skills in a safe environment in health care and healthcare education [11]. Simulation offers realistic learning activities based on clinical scenarios with a focus on developing skills, combining knowledge and skills, and transferring knowledge to practice [12]. The simulations may consist of several modalities, such as case studies, role-playing games, and simulation with technology [13], or utilizing technical equipment, simulated patients, professional patients, virtual environments, or a combination of these [12]. Simulation training typically exposes the learners to the problem-solving of severe, time-critical, and potentially fatal scenarios, such as resuscitation [14], trauma care [15], and surgery [16], as well as strategies for improved interprofessional collaboration in acute situations [5].

Shift of care and treatment from hospitals to primary care, increased prevalence of long-term conditions, and complex care requirements depend on collaboration between healthcare professionals in primary care who are more accustomed to collaborate within their profession, organization and sector [17]. Thus, primary care professionals, and healthcare students, do not necessarily have the skills, knowledge, and values needed to collaborate with the range of professionals they will meet during their professional work [18]. Previously reported primary care education studies include simulation scenarios for home visit preparation [19], home care and safety assessments [20], medication management [21], patient consultations [22], and end-of-life care [23].

Expanding simulation training to include common, sub-acute primary care scenarios offers the learners complex situations where they have time to assess, discuss, and collaborate to solve the problem. In particular, the development of shared treatment plans can work as a means to improve communication, coordination, and collaboration, consequently resulting in a more coherent plan for the patient [24]. These scenarios are typically not as time-critical and dependent on detailed algorithms or checklists as many acute-care scenarios. The outcome is dependent on the learners’ capacity to use their knowledge in practice, and to collaborate and expand on their clinical judgment together. Thus, such simulation may prepare the students for realistic and common clinical situations. Introducing simulation-based IPE with a focus on primary care scenarios can supplement traditional simulation approaches for developing the collaborative competence required to work in healthcare teams.

This paper describes the analysis of healthcare students’ interactions while exploring common, sub-acute patient scenarios in primary care situations, and aims to explore how healthcare students’ actions influence interprofessional collaboration and treatment plan identification.

### Theoretical perspective

This study adopts a socio-cultural perspective where knowledge and learning are constructed and co-created in interactions between participants, environments and artefacts (tools and objects) in a social practice [25, 26], herein “simulation” is the social context. Thus, learning is viewed as a result of participating in social activities and collaborating with others in a cultural context to solve mutual problems, produce outcomes, and gain insight. Learning is further defined as a developmental process outlined as the zone of proximal development (ZPD). ZPD refers to a development space for students’ collaborating with others through social interaction [26]. This view of learning is also in line with the aforementioned definition of IPE as learning “about, from and with” one another emphasize learning through interactions in a social context [1]. These premises are complemented by the following constructs: shared knowledge objects, productive interactions, active participation, and interaction trajectories [27]. The *shared knowledge objects* are viewed as the materialization and co-creation of knowledge that represents the goal to be pursued (e.g. learning outcome) and the material outcome to be achieved through the activity (e.g. simulation) [27];. We understand *productive interactions* as verbal and non-verbal communicative exchanges between the participants leading to the co-construction of the shared knowledge objects [28]. *Active participation* is understood as deliberate, joint, knowledge driven activities contributing to the shared goal [27]. The *interaction trajectories* are viewed as coherent sequences of productive interactions, which unfolds as moment-to-moment events over time [27].

## Methods

### Research design

We conducted an explorative, qualitative study with video recordings of healthcare students participating in primary care simulation scenarios. The unit of analysis in this study is the collaborative actions (verbal and non-verbal) in which the shared knowledge is produced. Such actions comprise speech, bodily behavior, artefacts, and environmental structures.

### Participants and setting

We recruited 27 healthcare students close to graduation, 10 of which were medical students (MS), eight were master’s students in advanced geriatric nursing (AGN), and nine were bachelor’s students in nursing (NS). The students were allocated into 10 groups, and two groups participated in the simulation each day. Table 1 presents details regarding the participants.

**Table 1:** Participant description

	Total ***N*** = 27	MS ***N*** = 10	AGN ***N*** = 8	NS ***N*** = 9
**Age**				
Mean (SD)	31 (9.4)	28 (3.4)	42 (7.8)	25 (6.7)
Min-Max	21-49	24-34	28-49	21-42
	N (%)	N (%)	N (%)	N (%)
Male	6 (22.2)	3 (30)	1 (12.5)	2 (22.2)
Female	21 (77.8)	7 (70)	7 (87.5)	7 (77.8)
**Prior simulation experience**				
Yes	22 (82)	8 (80)	6 (75)	8 (88.9)
No	5 (18)	2 (20)	2 (25	1 (11.1)
**Prior interprofessional simulation experience**				
Yes	7 (26)	2 (20)	2 (25)	3 (33.3)
No	20 (74)	8 (80)	6 (75)	6 (66.7)

The simulation took place at UiO:eColab, a research laboratory with two fully equipped healthcare offices/consultation rooms separated by a control room. We used a Laerdal SimMan® patient simulator [29]. The patient simulator presented clinical signs such as pulse, blood pressure, breath movements, and heart and lung sounds. The facilitators were present in the simulation room, and acted as the patient’s voice and supplemented responses not available through the simulator.

### Simulation scenarios

We developed two simulations with common, sub-acute patient scenarios from primary care situations. The scenarios were developed based on the assumption that caring for patients with complex problems is often beyond the expertise of any single profession [30]. The two scenarios had a shared introduction with an older patient staying at a nursing home following surgery for a hip fracture. Then, the patient developed symptoms of either a urinary tract infection or pneumonia. The simulation session started with a briefing about the room, available (technical) equipment, a reminder about confidentiality, and an introduction to the scenario. During the briefing, we also emphasized to the students that collaboration was important. The students were assigned to perform a clinical assessment, agree on a reasonable clinical problem or diagnosis, and develop a shared treatment plan during the simulation. Each simulation scenario lasted for 25 to 35 min (mean 31 min). The facilitator conducted a debriefing directly after the simulation. The students were asked not to reveal the content of the simulation until all the groups had participated in both scenarios.

### Data collection, analysis, and transcription methods

We collected data during 5 days in April 2019. Video recording was chosen to enable repetitive viewings of the dialogue and interactions by the project group [31]. Discrete placement of cameras and audio recorders in the ceiling, which were operated from the control room, minimized interference from technical equipment. The recordings were directly imported to a secure data storage facility at the University of Oslo (TSD), where only the project group had access. The facilitators took field notes during the simulation.

We used interaction analysis to guide our analysis of the data. Interaction analysis is a useful method to study the unfolding interactions in play during a social activity, including talk, non-verbal interactions, and material artefacts [31, 32]. Initially, the first author undertook a preliminary, comprehensive review to obtain an overview of the data and then created a timecoded content log of key events for all the videos, a total of 20 h. Secondly, after a substantive review of the data, we selected the students’ efforts to develop a treatment plan for the patient for further analysis. The treatment plan was viewed as a representation of the shared knowledge object co-created in the interactions and the communicative exchanges between the students. Furthermore, shared treatment plans are essential for efficient coordination of care and, thus, are an important part of interprofessional collaboration. In a third analytical step, relevant segments from the video recordings containing the development of the treatment plan were extracted for final analysis. Verbatim transcription of verbal and non-verbal behavior in the extracted segments was performed. Then the segments and associated transcripts were analyzed in depth, focusing on the teams’ interactions when they developed the treatment plan. The first author translated the transcripts to English. In the transcripts, shorter pauses are marked by brackets with punctuation representing seconds, concurrent talk is marked by double slashes at the start and end of an excerpt, and half sentences are marked with single slash.

### Ethical considerations

We informed the students about voluntary participation, confidentiality, video recording and data storage, and that only the project group had access to the recorded material. We obtained informed consent after giving oral and written information. The Norwegian Centre for Research Data approved the study (project number 60867).

#### Strategies to enhance rigor and trustworthiness in the analysis

The video recordings facilitated repeated review of the material, individually and in group work, by coauthors with different backgrounds, helping to ensure the legitimacy of our interpretations [33]. The first and last author were present for all of the simulations, while the remaining coauthors were present for one to 3 days, enabling familiarity with the material. The authors are nurses (LL, AM) and medical doctors (RBJ, EOR, AMB) working in research and education (e.g. teaching, curriculum planning, and simulation training). The authors also have experience from different healthcare settings, including primary care. This might produce unconscious preconceptions about the activity in the simulations; however, the authors’ experience may also facilitate awareness and understanding of what these situations entail. In the following transcribed extracts, extensive details are provided to make it possible to follow the talk and interactions, ensuring a high level of transparency [33].

## Results

The analysis revealed that the content and structure of the treatment plans varied between the groups and was influenced by the interactions and communication between the students. This led us to divide the material into two groups: *specific treatment plans* and *non-specific treatment plans*. A specific treatment plan had relevant, clearly defined clinical problem(s) with defined, related actions and interventions. A non-specific treatment plan either had several unspecified clinical problems or lacked defined problems entirely, and the actions and interventions were non-specific or nonexistent. Table 2 shows overall performance in creating a treatment plan during their first simulation.

**Table 2:** Overall performance in the 10 groups

Group	Participants	Overall performance in creating a treatment plan
1	MS, AGN, NS	Specific treatment plan
2	MS, AGN, NS	Non-specific treatment plan
3	MS, AGN	Non-specific treatment plan
4	MS, AGN, NS	Non-specific treatment plan
5	MS, NS	Non-specific treatment plan
6	MS, AGN, NS	Specific treatment plan
7	MS, AGN, NS	Non-specific treatment plan
8	MS, AGN	Specific treatment plan
9	MS, AGN, NS	Non-specific treatment plan
10	MS, NS, NS	Non-specific treatment plan

In the groups that engaged in productive interactions, and created a specific treatment plan, we observed a deliberate, collective strategy bringing multiple perspectives into the discussions. The interactions unfolded as coherent sequences where the students actively built on each other’s input in a joint effort. The students shared information and discussed, and their collaborative actions led to the emergence of new knowledge and progress of the shared treatment plan. In the groups that created non-specific treatment plans we observed circular discussions, with repetition of prior statements without clarifying the concepts. There were limited contributions and less active participation in the groups, which led to less development of shared knowledge, and ended with a non-specific treatment plan. We have selected unfolding interactions as examples from two groups that prepared a specific and non-specific treatment plan to illustrate the most typical interactions and co-creation activities observed in the simulations. In the following section, we present examples from Group 1 and Group 2 to help explain and visualize these interactions.

### Interactions and collaborative co-creation activities leading to a specific treatment plan

Group 1 consists of an MS, AGN, and NS and they are simulating a scenario with pneumonia. At the beginning of the simulation, they are sitting by the table in the office, with the AGN in the middle. The medical record lies open in front of the MS and AGN. There is a notepad with notes on the table in front of the NS. Everybody is looking at the medical record.

#### Establishing shared understanding

In the following excerpt, we meet Group 1 at the starting point of the planning phase (Table 3). Before this excerpt, they agreed on pneumonia and dehydration as tentative clinical problems for the patient.

**Table 3:** Excerpt 1 from Group 1

	Participant^**a**^	Verbal	Non-verbal
1.1	MS	Ehm (..) It (.) / I think that we at least can ehm, consider giving him some fluid. He had drunk a little, but //he//	Starts hesitantly, but continues to talk in a normal, clear tone. MS turns toward AGN at “I think”. MS alternates between looking at AGN and NS. Both look at MS. Uses questioning tone at “drunk a little.”
1.2	NS	//a coffee// was what he //said//	Confirms in an agreeable tone. NS nods.
1.3	MS	//yes//	Positive, confirmative tone. MS points to NS.
1.4	AGN	//mm//	Confirmatory sound. AGN reaches towards the medical record with right arm.
1.5	MS	He appeared a little dry (*dehydrated*)	Questioning, open tone
1.6	NS	mm	Light, confirmative tone
1.7	AGN	Yes, eh //he is on//	Clear, agreeable tone at first, then explanatory. AGN flips the medical record to the medication list. MS leans forward to look at the medication list.
1.8	NS	//let’s//see, shall we write down the plan? (..)	Questioning tone. NS takes the notepad and picks up a pen. Writes something in the notepad.
1.9	AGN	ehh (..) He is on diuretics, on Furosemide	Hesitant tone, while reading the medication list; turns it slightly towards themselves. Scratches face with right hand. Explanatory, friendly tone.
1.10	MS	Right	Confirmative, friendly tone

^a^*MS* medical student, *NS* Nursing student, *AGN* Advanced geriatric nursing student

In the excerpt, we see that the MS starts with an open question about fluid intake (Section 1.1), inviting the other participants to contribute. The invitation is accepted, with both the NS and AGN contributing. The NS writes on the notepad and suggests writing their plan down (Section 1.8). The AGN reads the medication list and explains that the patient is on diuretics.

The first notable finding in this excerpt is that this group’s interactions were aimed at creating a shared understanding from the start. By looking at the AGN and NS, talking in an open, questioning tone, the MS addresses both directly to assess their interpretation of the situation. The conversation between the participants is characterized by equal contribution, albeit in half sentences. Nevertheless, they elaborate spontaneously on one another’s input. The NS suggests writing down a plan in an attempt to structure the group’s knowledge. They continue to elaborate on one another’s suggestions, contributing to further clarification and specification of diagnostic tests and treatment.

#### Mobilizing mutual knowledge

Leading up to the following excerpt, the group had been discussing pain medication, which led the NS to suggest monitoring the patient’s pain. Both the MS and AGN agreed to this suggestion, with the MS exclaiming that it was a very good idea. The discussion continues in Table 4.

**Table 4:** Excerpt 2 from Group 1

	Participant^**a**^	Verbal	Non-verbal
1.11	MS	Do you have any other thoughts?	Friendly, questioning tone. Looks at AGN and then back at the medical record. NS writes.
1.12	AGN	(...) Ehh, let’s see what kind of forms there are here. I just thought about registering, in relation to (..) eh such (.) delirium and such //things//	AGN straightens and hesitates somewhat. Talks in a mild, explanatory tone and reaches for the box with forms. AGN lifts them up and goes through the pile of forms. MS and NS watch, leaning towards AGN. AGN continues to talk in an explanatory tone.
1.13	MS	Yes	Clear, agreeable tone
1.14	NS	mm	Light, agreeable tone
1.15	AGN	Ehm (...)	Hesitant sound uttered while AGN reads.
1.16	NS	Yes, the one there (..)	Quiet, suggestive tone. NS points to the form.
1.17	AGN	Yes, the one there (..) 4AT^b^	Cheerful, friendly tone. AGN shrugs a little at “4AT”, then smiles and laughs.
1.18	NS	Yes, right	Cheerful, friendly tone. NS smiles and brushes hair away from face with right hand.

^a^*MS* Medical student, *AGN* Advanced geriatric nursing student, *NS* Nursing student. ^b^4AT = refers to the rapid clinical test for delirium

As illustrated in this excerpt, MS actively addresses the AGN and invites the AGN to contribute in a friendly tone (Section 1.11). The AGN hesitates slightly and reaches for the forms. The AGN flips through the pile of forms and explains that registering delirium should be considered (Section 1.12). Then NS spots the form and points towards it (Section 1.16). The AGN confirms that the NS is correct in a cheerful tone (Section 1.17).

The excerpt presented in Table 2 illustrates that the MS actively seeks to obtain the AGN’s specific knowledge in an effort to elaborate on the treatment plan. The AGN gives an impression of familiarity with the use of different forms to assess delirium and takes initiative to look for a suitable form. This is picked up on by the others, with both the NS and MS leaning towards the forms as the AGN goes through them. The NS spots the form first, draws attention to it and gets confirmation from the AGN. In doing this, they utilize the tools and resources available in the room to expand on the joint knowledge development.

So far, these short excerpts show Group 1’s attempts to gain knowledge by using artifacts, and that they continue to build on one another’s statements and suggestions in order to co-create a coherent treatment plan.

#### Elaborating on and reframing the shared knowledge

Continuing the planning phase, the group has discussed the 4AT (a tool for delirium assessment) and National Early Warning Score (NEWS) forms. The MS expressed unfamiliarity with the 4AT and NEWS forms, and the AGN and NS both contributed in explaining the forms’ aims and usages. In the following excerpt (Table 5) Group 1 starts to conclude their treatment plan.

**Table 5:** Excerpt 3 from Group 1

	Participant^**a**^	Verbal	Non-verbal
1.19	NS	So, to conclude	Clear, friendly tone. NS points to the notepad when mentioning concluding.
1.20	MS	mm	Confirmative sound
1.21	NS	We administer 1 l of Ringer Acetate slowly now	NS continues to summarize in a clear, friendly tone and actively points to the notes, tracks the notes, and looks down at the notepad.
1.22	MS/AGN	mm	Confirmative sound. MS and AGN nod, both hold their attention towards NS.
1.23	NS	Intravenously. We send a urine sample for cultivation. Eh (.) see if we can get a nasopharynx test	Still clear, friendly tone. Hesitates slightly and looks from MS to AGN when mentioning nasopharynx. Seems open for input and/ or questions.
1.24	AGN	mm	Confirmative sound. AGN nods.
1.25	MS	Yes	Confirmative tone. MS nods.
1.26	NS	Eh, and then we try to monitor the pain with VAS scale^b^ if we can manage. Then we can see (…)	NS hesitates a little again, continue to track the notes and talks in a slightly questioning tone. Looks at AGN when mentioning VAS. NS shrugs at “if we can manage” as if unsure if it will work. NS gestures with right arm at “can see.”
1.27	AGN	There are different types //of pain scales yes//	AGN looks at NS. Confirmative, explanatory tone. Nods. NS and MS hold their attention towards AGN.

^a^*NS* Nursing student, *MS* Medical student, *AGN* Advanced geriatric nursing student. ^b^VAS scale refers to visual analog scale

This excerpt shows that the NS initiates the summarization of the treatment plan (Section 1.19). The NS summarizes in a clear, friendly tone, while actively pointing to the notes on the notepad (Section 1.21). The MS and AGN look attentive towards the NS and contribute with clear, confirmative sounds (Section 1.22). The NS appears unsure about the possibility of monitoring pain with the suggested form, hesitating and talking in a questioning tone while looking at the AGN (Section 1.26), prompting the AGN to explain (Section 1.27). After this excerpt, they continue to discuss different pain scales and then the NS continues to summarize the plan. The MS and AGN contribute with input to make the plan more specific.

Here, the students attempt to construct an overview by summarizing their existing knowledge, initiated by the NS. Through the structuring and reframing of the proposed treatment plan, the group members collectively arrive at a better understanding of how to treat the patient. The way in which the students continuously dealt with uncertainty by explaining and elaborating upon the concepts in question during the planning shows that they are attentive towards one another and actively seeking joint knowledge. This example is illustrative of interactions that lead to a concrete and specific treatment plan.

### Interactions and collaborative co-creation activities leading to an unspecific treatment plan

Group 2 has the same composition as Group 1, with an MS, AGN, and NS, and they are also simulating the scenario with pneumonia. At the beginning of the simulation, they are sitting by the table in the office, with the MS in the middle. The medical record lies open in front of the MS. A notepad with notes is on the table in front of the AGN. The AGN and NS look at the MS.

#### Identifying collective uncertainty

In the excerpt presented below, we meet Group 2 approximately 1.5 min into the planning phase. Prior to this excerpt, the MS expressed an intention to admit the patient to hospital due to confusion. The MS also suggested administering intravenous fluids and bladder scan at the nursing home. The AGN contributed with agreement and repeated the MS’s statements. The NS said nothing except in a response to the MS about the amount of urine output. The planning continues in Table 6.

**Table 6:** Excerpt 1 from Group 2

	Participant^**a**^	Verbal	Non-verbal
2.1	MS	I also want to call (.) / Call to confer about him at least	Light, hesitant tone. Looks down at the medical record.
2.2	AGN	mm	Confirms in a quiet tone. AGN looks at MS and nods. NS says nothing, but puts left elbow on the table and lays chin in hand.
2.3	MS	At ehm (..) at the hospital. Since we have no information as to why he should suddenly become deli / become disoriented	MS starts to speak in a slightly hesitant tone. MS turns head and makes eye contact with AGN at “information.” Stronger, more confident tone, with a little hesitation at the end. Shakes head.
2.4	AGN	No, we do not have any	Light, agreeable tone. AGN waves left hand over notes while speaking.
2.5	NS	Mm	NS nods and utters a non-committal sound.
2.6	AGN	Completely clear	AGN looks down at the notepad, moves it a little and speaks in a light, friendly tone.
2.7	MS	No	Clear, agreeable tone.
2.8	AGN	Clear reasons for //what it could be//	MS and AGN talk at the same time, look at each other. Both speak in friendly tone.
2.9	MS	//Right//	

^a^*MS* Medical student, *AGN* Advanced geriatric nursing student, *NS* Nursing student

In this excerpt, the MS starts speaking hesitantly and suggests conferring with the hospital, since they are unsure of why the patient is disoriented (Sections 2.1 and 2.3). The AGN confirms the MS’s statements and agrees with the MS (Sections 2.4 and 2.6). Then, the AGN indicates the lack of information by waving a hand over the notes. The NS does not contribute with any relevant content.

The excerpt above illustrates how the MS acknowledges their collective lack of knowledge as to why the patient is disoriented. At once, the AGN contributes with agreement and repetition of the MS’s statement. Thus, they have agreed on their mutual knowledge about the patient’s condition: the fact that they do not understand it. Voicing this collective uncertainty has the potential to strengthen the collaborative effort to explore the problem at hand. The excerpt also shows that they have the opportunity to utilize other resources to solve the problem by conferring with the hospital. However, when we observe the students’ subsequent actions, it is obvious that they do not act on their prior statements.

#### Insufficient elaboration of concepts

Before the next excerpt, the MS talked about the operation wound and contacting the patient’s relatives. The AGN mostly replied in a confirming tone without further contribution. The NS went to the patient to perform a practical assignment (bladder scan). Then, the MS mentioned frequent monitoring at the nursing home in case admitting the patient to the hospital takes time, if they accept to admit him at all, still without any elaboration or contribution from the AGN or NS. Table 7 shows the excerpt from the continuing simulation.

**Table 7:** Excerpt 2 from Group 2

	Participant^**a**^	Verbal	Non-verbal
2.10	MS	Ehm, mm (...) Let us see, is there anything we have not thought of? I think if I can admit him to hospital then there is no reason to make a long plan, but it	Hesitant sound at first, then use a questioning, open tone. MS straightens, puts hand to face. Explanatory, friendly tone. MS flips through the medical record, gestures with left hand at “long plan.” AGN looks up from the notes and puts hand to face. NS still leaning with chin in right hand.
2.11	AGN	No	Light, confirmative sound. AGN looks at the medical record.
2.12	MS	but we might need to have a plan if they do not want to accept him (..) Even though they cannot really refuse. But eh	Questioning, open tone at first, then a light, cheerful tone when saying that they cannot refuse. MS smiles and flips the medical record.
2.13	AGN	Should we wait until (…) if the time comes and they won’t accept him, then make a plan	Questioning, light tone. Looks at MS questioningly and hesitates a little at “if the time”. MS puts right hand to chin and turns towards AGN. NS straightens up, then resumes the same position as before (leaning chin on hand).
2.14	MS	Yes. We could have a tentative treatment plan in case this suddenly	Clear, confirmative tone at “yes,” then explanatory. MS nods, looks at the medical record.
2.15	AGN	mm	Light tone. AGN nods, then leans on the table with both hands. Looks towards MS.
2.16	MS	improves spontaneously, eh	Explanatory, light tone. MS gestures briefly with right arm at “improves.”
2.17	AGN	Yes	Light, confirmatory tone. AGN nods.
2.18	MS	with fluids and (.) better pain relief	Explanatory, light tone. Looks at the medical record.

^a^*MS* Medical student, *AGN* Advanced geriatric nursing student, *NS* Nursing student

This excerpt shows that the MS initially aims for a discussion with the AGN and NS by asking if there is anything they have not thought of. However, the MS quickly returns to the concept of admitting the patient to hospital yet again (Section 2.10). Then the MS changes direction towards making a plan if the patient is not admitted (Section 2.12). The AGN picks up this statement and hesitantly suggests waiting to make a plan until they know if the patient will be admitted (Section 2.13). Initially, the MS agrees, but then explains that they should have a tentative treatment plan prepared if fluids and pain relief improves the situation (Sections 2.14, 2.16 and 2.18). The NS says nothing.

In this excerpt, we see that the group seems inclined to return to possible hospital admittance as the main intervention for the patient. Although this concept repeatedly occurs in the simulation, they continue to add other potential interventions sporadically. The discussion, however, often stops at the point of mentioning an intervention, such as administering fluids, without further elaboration of why or how. In turn, they do not arrive at a mutual understanding of the concepts and the conversation circles back to the hospital. The talk is mostly driven by the MS, who appears unsure of what to do. They have not stated any tentative clinical problems for the patient, which consequently seems to make it difficult to refine, elaborate on, or conceptualize a treatment plan.

#### Inability to bring concepts to action

Leading up to the following excerpt, the MS has talked about intravenous fluid, possible constipation, and optimizing pain medication. The AGN mentioned coughing, but the MS dismissed it because of clear lungs when auscultating. The MS commented on fluids again and monitoring fluid intake and output to optimize the patient, if not admitted. The MS then asked if anything had been forgotten, and the AGN mentioned nutrition. The discussion continues in Table 8.

**Table 8:** Excerpt 3 from Group 2

	Participant^**a**^	Verbal	Non-verbal
2.19	MS	I completely agree with that. So if it calms down now then we (..) / we must try to get him to eat. Right now when he is / want to clarify his condition a little more first	Clear, agreeable tone at first, then explanatory, friendly tone from “So if.” Hesitates a bit, but continues in explanatory, friendly tone. MS gestures slightly in front of chest at “get him to,” and looks down at the medical record.
2.20	AGN	Mm	Light, agreeable sound. AGN looks at MS.
2.21	MS	eh (..) If he is going to go in for a revision of the wound then it is/ foolish / a little foolish if he is not fasting. So it all really depends on //on what//	Hesitant start, then explanatory, friendly tone. All three look at the medical record. Friendly, cheerful tone. MS smiles and chuckles.
2.22	AGN	//depends on whether he// is admitted or not	Friendly, cheerful tone. AGN also smiles and chuckles.
2.23	MS	Yes	Cheerful and confirmative tone from both, both continue to smile
2.24	AGN	Yes	

^a^*MS* Medical student, *AGN* Advanced geriatric nursing student

This excerpt shows that the MS acknowledges the suggestion from the AGN regarding nutrition (Section 2.19). However, in the same section, the MS explains why it is important to clarify the patient’s condition first, because wound revision in the hospital requires fasting (Section 2.21). Thus, the MS rejects the contribution from the AGN due to the possible necessity for surgery. At this point, the MS starts to admit that hospitalization is uncertain (still Section 2.21), and is interrupted by the AGN who adds that everything depends on admittance or not (Section 2.22). The facilitator ends the simulation.

The excerpt above illustrates that the group did not progress in their development of the treatment plan. They were not able to generate concrete ideas or further elaborate on the concepts they shared. Any attempt to start a joint discussion about the treatment plan and materialize these ideas is stopped by their inability to define the patient’s clinical problems. The planning phase goes around in circle, with wanting to confer with the hospital about the patient’s confusion, then admitting him without any clear indication as to why, then suggesting to make a tentative treatment plan in case he is not admitted, and then talking about admittance due to wound revision. The group seems to have knowledge of the situation in practice, but appears to have difficulties conceptualizing, elaborating on and refining a treatment plan.

#### Summary of findings

**Fig. 1:**
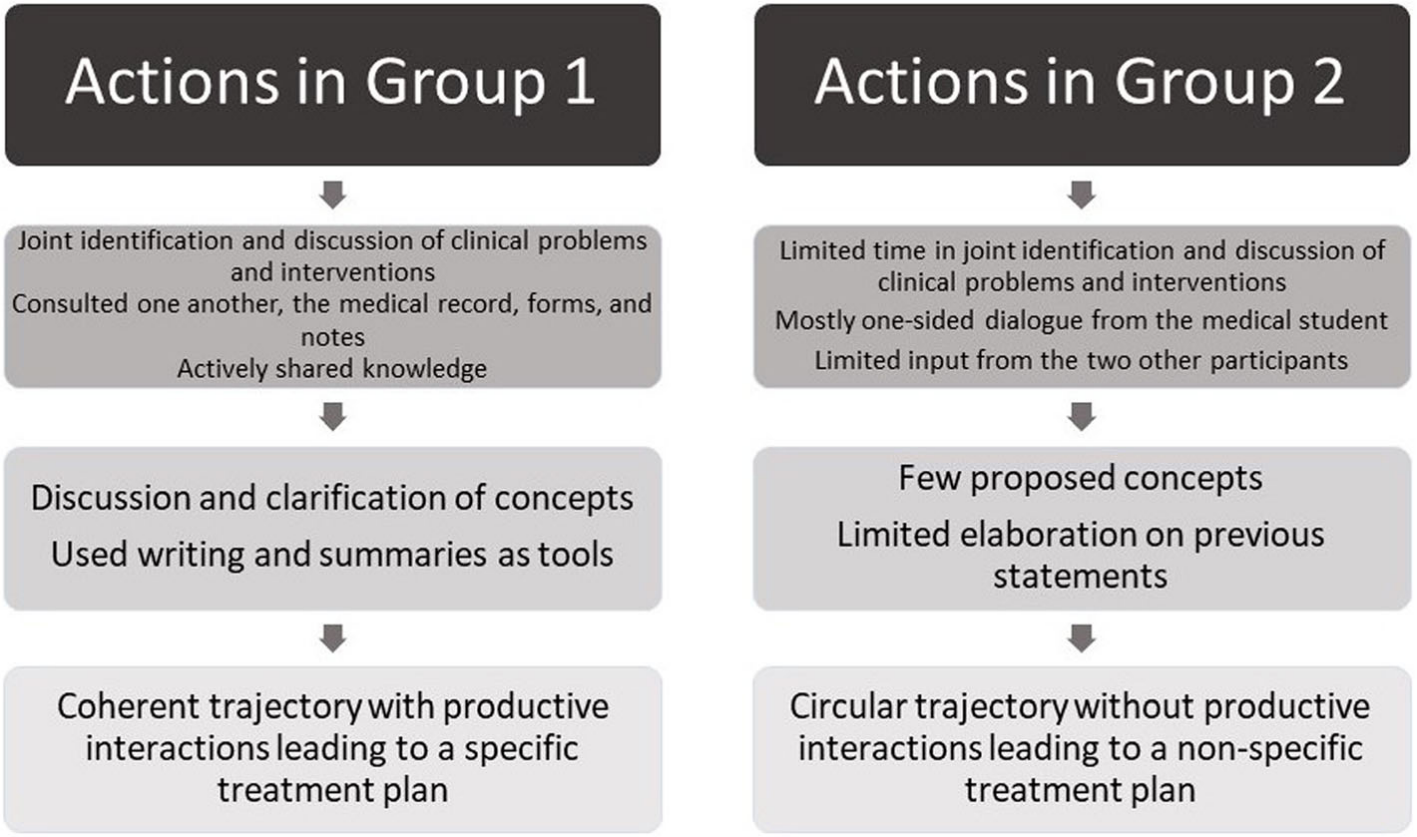
Integration of findings

The interaction and co-creation process ranged from discussions, efforts to structure knowledge, and use of tools in Group 1, to repetition of prior statements without further elaboration in Group 2, as illustrated in Fig. 1.

In summary, we found variation in how the groups engaged in interactions, the paths they followed and the quality of their shared knowledge object, the treatment plan.

## Discussion

The analysis showed that the development, content, and structure of the shared treatment plans were influenced by the interactions within the group. The groups that managed to engage in productive interactions in a coherent interaction trajectory developed a more comprehensive and specific treatment plan than the groups where the interactions were less productive.

### Productive interactions

We identified that several productive interactions occurred during the collaborative work, exemplified by Group 1, such as joint discussion and elaboration of concepts. The students in Group 1 mediated their actions by utilizing the artefacts and tools available to them [25]. Through speech, the students verbally invited each other to participate using friendly, open tones. By looking directly at one another and being attentive towards the ones speaking, they encouraged each other to participate through gaze and body language. This exemplifies some of the productive interactions mediating their actions in an effort to understand their task, and to construct and refine the shared understanding of the patient’s problem.

In contrast, Group 2 spent limited time in joint identification and discussion of concepts and were less productive in mediating the artefacts and tools available to them. Although talking in friendly tones, the verbal contributions were mainly confirming sounds or repetition of statements, instead of suggestions or elaborations. They had sporadic eye contact, but most of their gaze faced their notes or the medical chart. Consequently, they did not manage to utilize available productive resources to advance in their conceptualization of the treatment plan.

Our results illustrates that the groups where the students participated actively in sharing knowledge, practice and experiences managed to engage in productive interactions and refine the treatment plan in a collaborative effort. ZPD can be seen as a process where the student’s performance is co-constructed in interaction and collaboration with others [26]. The goal is to provide a developmental space for the students where the learning situations stretch the students’ capabilities towards the edge of their ZPD, without pushing them too far. Interprofessional learning activities should equip students with the necessary competencies to participate actively and share knowledge to stretch their expansion in the ZPD [26]. Thus, educators have a vital role in developing collaborative learning activities that stimulate and support the students reaching toward higher levels in their ZPD [34]. For the students to be able to actively participate and engage in productive interactions and create expansion in their ZPD, however, they have to be able to make sense of the actions played out in the simulation. Making sense of a situation relates to understanding and acting on signs and actions in the activities, but also being able to draw on available resources [35]. As such, being able to develop a specific treatment plan can be seen as a result of the productive interactions between the participants in the simulation, intertwined with the capacity to make sense of the scenario at play. Consequently, when developing IPE scenarios with the aim to expand the students’ activities within the ZPD, educators need to consider the level of realism - often referred to as fidelity - and difficulty needed to optimize learning opportunities [36, 37]. Enhancing simulation fidelity is described by Dieckmann and Ringsted [36] as optimizing the educational value of the simulation, and is not solely about maximizing difficulty or use of simulation equipment. They highlight that slowing down the physiological deterioration of the patient in a simulation might be a solution to give the learners more time to react. If the learning activity is perceived as too demanding, it might be impossible for the students to make sense of the situation, compromising advancement in their ZPD. Consequently, leading to missed learning opportunities and a negative experience for the students. In contrast to healthcare professionals, students attend simulation to develop skills they not yet have fully acquired in a more or less unfamiliar practice situation [38]. The students’ ability to make sense of the situation seems to be connected to how far they have progressed in their education, but also what clinical practice they have had. As such, it is a delicate balance for educators designing interprofessional collaborative simulations, to clarify what technical skills and which level of clinical deterioration is necessary to expand the students’ ZPD.

Our scenarios presented the students with sub-acute situations from a nursing home, and were not dependent on the students having advanced technical skills. However, we acknowledge that they might have perceived the scenarios as complex due to an atypical presentation of symptoms and lack of algorithms to follow. Still, since the simulation scenarios comprised common primary care situations and a slow pace, the students had time to assess, plan, and talk together to solve the problem. The scenarios seemed to facilitate the development of the students’ collaborative competencies, and expand their ZPD, as long as the student managed to make sense of the activities. Our study highlights the feasibility of sub-acute primary care simulation scenarios to teach communication and teamwork in situations where an accurate diagnosis and decisions about treatment may be difficult to make. After completing the simulation together, the students may be better prepared to participate and contribute in this type of scenario in the future. Through that process, the students ZPD will have been expanded. The next time the students participate in simulation, they should manage a higher level of difficulty, and further develop their ZPD.

### Interaction trajectories

Our results revealed two distinct trajectories when developing a shared treatment plan. By contrasting two groups, we have illustrated how the co-construction process of shared knowledge can take different routes and lead to different results. Ideally, the groups should follow a coherent interaction trajectory, as seen illustrated in Group 1, where the elaboration on previous statements, use of available resources, and interactions created possibilities for the development of the shared knowledge object [39]. However, as exemplified by Group 2, some of the groups remained in a circular trajectory with few proposed concepts and limited elaboration on previous statements, which affected the development of a treatment plan. For educators, zooming in on why some of the groups had difficulties establishing a functional way of interacting, is important to enhance future IPE. One way of addressing the *why* is to look at the IPE definition where learning “about, with and from”, is a cornerstone. Still, healthcare students are educated in professional silos [2] and there are common assumptions that students are exposed to interprofessional collaboration during clinical practice [1]. The students may not have had the opportunity to be involved in interprofessional collaboration and thus need more knowledge *about* other professions and *about* their own role in the collaboration. We exposed the students to collaborative activities where they had to develop a shared treatment plan for the patient without providing instructions on how the task could be completed. Expansion of the students’ ZPD requires active participation and some students might not feel comfortable with active participation if they are insecure of *how* to participate. In our study, only seven of the 27 students had prior interprofessional simulation experience and we do not know which groups these students were allocated to. Neither do we know anything about their experience from clinical practice. Limited previous experience might have affected their ability and willingness to take an active part in the scenarios. In addition, the students had not met prior to the simulation activity. They did spent some time in informal conversation in the groups to get to know each other before the simulation started. Nevertheless, a failure in creating a specific treatment plan could be explained by the fact that the students had just met, and thus were a bit hesitant in their interactions.

In our results, exemplified by Group 2, we saw that some students seemed to entrust identifying and generating knowledge to the medical student. It can be a complex task for students to know how to engage in interactions that lead to concrete ideas and to further elaborate on those ideas to develop a shared knowledge object [28]. The students have to draw on experience and knowledge from their education and clinical training during the simulated scenarios. Thus, healthcare students have different profession-specific knowledge, in addition to personal values and beliefs, which in turn could affect the communication and shared knowledge development. Participating in these learning activities might remain complex for some students, especially if they are unsure of their own competence or their role in the scenario. In addition, presumed power relations and hierarchical structures, where the medical doctor is seen as the expected leader, may also be a barrier to participate [40]. As such, some of the students might decide to listen and learn *from* the other students, rather than contribute actively in learning in interaction *with*. This would limit their own contributions, but also the other students’ possibility to advance in their ZPD. Making the students feel safe and confident in the learning situation can foster confidence in the students own role and willingness to participate in a team [6], and consequently contribute to co-construction of knowledge. Offering learning activities with a non-hierarchical structure may create a safe environment for teaching interprofessional collaboration for students in primary care settings, where the team is greater than the sum of its parts [41]. We believe that our scenarios have potential to be a safe way to develop collaborative competence as everyone’s knowledge is essential in solving the problem and different perspectives are valued and necessary to create the treatment plan.

### Implications for conducting IPE

As we have sought to understand how to organize student activities supporting interprofessional learning, we found that productive interactions and coherent interaction trajectories are important aspects for training interprofessional collaboration. We have contrasted and compared two groups to visualize these interactions and trajectories. Our results indicate that the groups with coherent interaction trajectories managed the duality of defining and solving the immediate problem and preparing for future care in collaboration. Those groups with circular trajectories may miss important opportunities for interprofessional learning.

When planning and implementing IPE, educators should have strategies available to prevent or detect the problems that the students encounter to help them move beyond circular trajectories during the simulation. Understanding the students’ current knowledge and capabilities, and discovering emerging problems, can help educators determine how to organize or change the simulation so that the students advance in their ZPD. Thus, educators, and especially the facilitators directly involved when the simulation is in progress, need to be flexible in their roles and adapt to the students’ needs [42]. In retrospect, we acknowledge that we in a way contributed to the assumption of students being able to collaborate without instruction or tools since we did not provide any instructions on how to collaborate. This lack of instruction or pre-briefing could explain some of the reasons why there was a difference in trajectories. We had a facilitator present in the room with the students to provide the patient’s voice and supplement responses the simulator could not, presenting ample opportunity for in-scenario instruction. In-scenario instruction is seen as essential for bridging the gap between a patient simulator and a real patient, but can also be used to give information as a response to the participants’ questions or actions [37]. On that account, we believe it prudent to consider using in-scenario instruction when groups are stuck in a circular trajectory. Those instructions could be aimed at helping students to structure concepts, elaborate on ideas, or identify key concepts for further discussion. This will enhance the potential for teaching interprofessional collaboration and contributing to the management of IPE in healthcare education.

Another possibility is to present strategies for collaboration, interaction trajectories and shared knowledge development in the preparation and briefing sequences before the simulation scenarios or add an introductory IPE course before the simulations. This might better prepare the students for interprofessional collaboration as they are presented with strategies on how to collaborate.

Reflecting on the simulation experience is seen as a cornerstone for students to reconstruct their experience into learning [42]. As such, the debriefing sessions provide ample opportunities for reflection on how the students collaborated. Students are often more concerned with their individual actions and if they managed to identify the solution to the medical problem [38]. The facilitator’s role in the debriefing process is to challenge the team to reflect on how they collaborated, how the different team members contributed and to evaluate each other’s contributions. The simulation setting, including debriefing, allows students to share their profession-specific knowledge and skills with one another, with the potential to expand the learning opportunities for each student and build trust in the clinical competence of other professions.

Despite IPE literature listing interprofessional collaboration and communication as competencies to achieve through IPE [43], it is less clear how the students can develop these competencies. Similarly, most healthcare education programs are profession-specific, and constrains such as schedules, actual space capacity, teacher resources, and economy may affect the educators’ possibilities to facilitate for IPE [7, 8]. How to overcome these barriers is a constant struggle for educators. Our scenarios do not require high-tech equipment in the educational facility or practice, nor specialized technical competencies from the facilitators. We used a patient simulator in this study, but this is not necessary, as a fellow student, educator, or healthcare personnel could easily play the role of the patient. Thus, our simulation scenarios are feasible to use on-site in an education facility or in practice with minimal equipment and resources. This might contribute to reduce some of the economic barriers toward IPE as the educational facilities do not have to buy expensive equipment or educate highly specialised facilitators.

### Limitations

There are several limitations to our study. We acknowledge that the students agreeing to participate might be the most receptive to IPE and simulation. Reasons given for not participating were lack of time, not granted leave of absence from clinical practice or work, or feeling uncomfortable with video recordings. We tried to avoid non-participation by emphasizing that the interactions between the participants were of interest and not their technical skills, and that only the project group would view the actual recordings. We also provided letters to deliver to leaders and educators to help the students get approved absence. The simulation was completed in 1 day, which also minimized absence from work or practice. Our study has a small sample size, as is typical of a qualitative study [33]; thus, our findings are based on a small number of recorded simulations. However, these recordings comprise a large amount of data, enabling detailed study of the interactions and activities within the groups. We have also chosen to present and contrast representative sequences from two groups relevant to the aim of the study. When using interaction analysis, the analysis is based on the researchers’ interpretations of the collaborative actions. The students themselves were not invited to comment on their own achievements or our interpretations, and we acknowledge that they might interpret or explain the situations differently from us.

There are also limitations when using video recordings, as the participants might change their behaviour due to the camera. However, in the research facilities where we conducted the simulations, the cameras and audio equipment were discretely placed in the ceiling, minimizing the interference. We used a patient simulator as the patient, which might induce lack of realism, as the simulator does not have facial expressions or the ability to respond. To enhance realism, the facilitators were present in the simulation room, acting as the patient’s voice and offering responses not available through the simulator. This in itself could also be a limitation to the realism of the situation. However, informal student feedback suggests that the facilitator added to the realism by acting as an older patient when communicating with them as the scenario played out. The students were not given any concrete tools on how to achieve interprofessional collaboration before the simulation, which might have limited their ability to maximize collaboration. We have suggested adding these strategies to the briefing or implementing an introductory IPE course.

## Conclusions

The present study of simulation in common, sub-acute patient scenarios in primary care situations illustrates that what seemed to characterize the groups engaging in productive interactions was a deliberate, collective strategy bringing multiple perspectives into the discussions in a coherent trajectory. For the students to actively participate and engage in productive interactions, and advance in their ZPD, they have to be able to make sense of the simulation. Use of in-scenario instructions might be a prudent way to help students to move out of non-productive trajectories and promote collaboration. Overall, the student activities in our scenarios show the potential for practicing interprofessional collaboration and adding simulations of sub-acute primary care scenarios as an area of importance in teaching communication and teamwork in complex situations. Therefore, we suggest that educators planning and developing interprofessional simulated scenarios should include common, sub-acute primary care situations. To further develop IPE strategies, research should expand on the interactions and collaborative efforts when people are learning through interprofessional simulation.

## Data Availability

The datasets generated during and/or analyzed during the current study are not publicly available due to data corpus still being subject to analysis but are available from the corresponding author on reasonable request.

## Abbreviations

IPE: Interprofessional education
MS: Medical student
AGN: Master’s student in advanced geriatric nursing
NS: Bachelor’s student in nursing
ZPD: Zone of proximal development
